# PREditOR: a synthetic biology approach to removing heterochromatin from cells

**DOI:** 10.1007/s10577-016-9539-3

**Published:** 2016-12-06

**Authors:** Oscar Molina, Mar Carmena, Isabella E. Maudlin, William C. Earnshaw

**Affiliations:** Wellcome Trust Centre for Cell Biology, University of Edinburgh, Edinburgh, EH9 3BF UK

**Keywords:** Heterochromatin, Chromosome segregation, Centromeres, Mitosis

## Abstract

It is widely accepted that heterochromatin is necessary to maintain genomic stability. However, direct experimental evidence supporting this is slim. Previous studies using either enzyme inhibitors, gene knockout or knockdown studies all are subject to the caveat that drugs may have off-target effects and enzymes that modify chromatin proteins to support heterochromatin formation may also have numerous other cellular targets as well. Here, we describe PREditOR (protein reading and editing of residues), a synthetic biology approach that allows us to directly remove heterochromatin from cells without either drugs or global interference with gene function. We find that removal of heterochromatin perturbs mitotic progression and causes a dramatic increase in chromosome segregation defects, possibly as a result of interfering with the normal centromeric localization of the chromosomal passenger complex.

**Electronic supplementary material:**

The online version of this article (doi:10.1007/s10577-016-9539-3) contains supplementary material, which is available to authorized users.

## Introduction

Eukaryotic genomes are organized as a spectrum of global chromatin states with differing epigenetic profiles. The first of these to be identified in interphase nuclei as regions of compacted and decompacted chromatin were termed heterochromatin and euchromatin (Bannister and Kouzarides 2011). Heterochromatin is a transcriptionally repressive chromatin state that can be either facultative or constitutive (Oberdoerffer and Sinclair 2007). The former is a transient epigenetic state found at promoters that changes in response to the environment and during development to establish tissue-specific gene expression and differentiation. Constitutive heterochromatin remains compacted permanently throughout cell differentiation and in different cell types. Most constitutive heterochromatin is found at pericentromeric regions, although it can also be found at other sites, including telomeres (Saksouk et al. 2015) and the long arm of the Y chromosome in mammals.

Centromeres, defined cytologically as the primary constriction of mitotic chromosomes, are the loci that direct chromosome segregation during cell division (Fukagawa and Earnshaw 2014). Human centromeres contain long stretches of non-coding alpha-satellite DNA organized in high-order repeats (Aldrup-Macdonald and Sullivan 2014). Centromeres can be divided into two major compartments, the core centromere of “centrochromatin” (Sullivan and Karpen 2004) and the pericentromere. Centrochromatin nucleates the assembly of the kinetochore, a multi-protein complex that binds microtubules and directs chromosome segregation (Fukagawa and Earnshaw 2014). Centrochromatin is characterized by the presence of nucleosomes containing the centromere-specific histone H3 CENP-A (Earnshaw and Migeon 1985) interspersed with nucleosomes containing canonical histone H3 bearing epigenetic modifications associated with transcriptionally active chromatin, such as H3K4me2 and H3K36me2 (Sullivan and Karpen 2004; Bergmann et al. 2011; Fukagawa and Earnshaw 2014). Centrochromatin is flanked by pericentromeric heterochromatin distinguished by the presence of characteristic histone modifications, including histone H3 trimethylated on lysine 9 (H3K9me3), which binds heterochromatin protein 1 (HP1) and histone H4 trimethylated on lysine 20 (H4K20me3) (Allshire et al. 1995; Ekwall et al. 1995; Bannister et al. 2001).

Diverse functional roles have been attributed to pericentromeric heterochromatin. These include facilitating sister chromatid cohesion by recruiting and retaining cohesin complexes in metaphase (Bernard et al. 2001; Nonaka et al. 2002; Yamagishi et al. 2008; Gartenberg 2009), suppressing inappropriate homologous recombination between repetitive DNA elements (Peng and Karpen 2007) and kinetochore maintenance. Indeed, euchromatin-heterochromatin boundaries have been suggested to be preferred sites for kinetochore formation in yeast and *Drosophila melanogaster* (Folco et al. 2008; Olszak et al. 2011).

Despite disagreements regarding its functions (Bernard et al. 2001; Koch et al. 2008), the importance of proper regulation and maintenance of pericentromeric heterochromatin is suggested by the finding that knockout mice for the H3K9-specific methyltransferase Suv39h1 show increased chromosomal instability (CIN), embryonic lethality and are prone to tumour formation (Peters et al. 2001). Furthermore, cancer cells with lower levels of pericentromeric heterochromatin show CIN (Slee et al. 2012). However, the precise role of heterochromatin in regulating proper chromosome segregation remains unclear.

Epigenetic regulation of chromatin can be described as a series of consecutive steps in which an *E* *ditor* (writer or eraser) makes or removes a *M* *ark* on a chromatin protein. A *R* *eader* can either recognize this *M* *ark* or cease to do so if the mark is removed. The binding of the *R* *eader* establishes a *C* *hromatin state*, such as euchromatin, heterochromatin or centrochromatin. We refer to this as an E → M → R → C pathway. Here, we present a novel synthetic biology approach called PREditOR (protein reading and editing of residues) to dissect and manipulate E → M → R → C pathways and analyse their functional outcome.

In order to study the role(s) of heterochromatin on chromosome segregation, we designed a PREditOR strategy that allows us to remove heterochromatin without drug treatments or global protein knockdowns. Our studies reveal that heterochromatin removal from pericentromeric regions leads to chromosome segregation defects as a result of disruption of kinetochore structure, chromosome passenger complex delocalization and decreases in centromeric stiffness during mitosis.

## Material and methods

### Expression constructs

The SUV39H1ΔSET-EYFP constructs were obtained as follows. The chromodomain of SUV39H1 was amplified from a custom made cDNA library from HeLa cells and cloned into the NheI and AgeI restriction sites of the pYIP-EYFP vector (Bergmann et al., 2011) generating SUV39H1ΔSET-EYFP, which contains 3′ attL and attR sites for Gateway cloning (Fisher Technologies). Full length JMJD2D was PCR amplified from our cDNA library using the oligonucleotides JMJD-Fw (5′-caccatggaaactatgaagtc −3′) and JMJD-Rv (5′- ttaaacgggcacagg-3′). The PCR product was used for gateway cloning following the manufacturer’s instructions (Fisher Technologies), to generate the construct pYIP-SUV39H1ΔSET-EYFP-JMJD2D^WT^, which express this fusion protein from a CMV promoter and confers resistance to puromycin. To generate the D195A mutant of JMJD2D and the W64AY67A double mutant of SUV39H1ΔSET, the pYIP- SUV39H1ΔSET-EYFP-JMJD2D^WT^ construct was subjected to site-directed mutagenesis using the QuikChange II kit (Stratagene).

### Cell culture, transfections and drug treatments

HeLa cells were maintained in DMEM supplemented with 5% FBS (Invitrogen), 100 U/ml penicillin G and 100 μg/ml streptomycin sulphate (Invitrogen). Cells were grown at 37 °C in 5% CO_2_ in a humidified atmosphere. Transfections were performed using Xtremegene-9 (Roche) following the manufacturer’s instructions. In brief, for transfections of cells growing in 24-well plates on polylysine-coated glass coverslips, transfection complexes containing 3 μl Xtremegene-9 reagent and 1 μg plasmid DNA were prepared in 100 μl OptiMEM (Invitrogen). After 20 min of incubation at room temperature, 25 μl of transfection complexes was added drop-wise in each well. After 24 h, transfected cells were selected adding 2 μg/ml of Puromycin (Sigma) and grown for 24 additional hours before fixation.

For RNAi treatments, HeLa cell transfections were performed using Polyplus jetPRIME (PEQLAB, Southampton, UK) with the indicated amounts of siRNA oligos and 500 ng of plasmid DNA. After 24 h, fresh DMEM was added and cells were maintained for 24 additional hours before fixation.

### Indirect immunofluorescence and microscopy

Indirect immunofluorescence staining of cells fixed in 2.6 to 4% Formaldehyde/1xPBS was performed following standard procedures. The following antibodies were used: rabbit anti-H3K9me3 (abcam ab8898, 3% formaldehyde, 1/500), mouse anti-HP1α (Millipore MAB3584, 2.6% Formaldehyde, 1/1000), rabbit anti-CENP-C (R554, 2.6% formaldehyde, 1/500), rabbit anti-CENP-B (WCEB4, 2.6% formaldehyde, 1/500), mouse anti-HEC1 (abcam AC3612, 2.6% formaldehyde, 1/1000), mouse anti-Tubulin (Sigma B516, 1/2000), rabbit anti-SGO1 (A. Losada, 4% formaldehyde, 1/1000), rabbit anti-SMC2 (A. Losada, 4% formaldehyde, 1/1000), rabbit anti-Pericentrin (abcam AB4448; 4% formaldehyde, 1/500), rabbit anti-Survivin (Cell Signalling, 4% formaldehyde, 1/400) and rabbit anti-INCENP (Cell Signalling P240, 4% formaldehyde, 1/500). Fluorophore-conjugated secondary antibodies were purchased from Jackson Labs.

Microscope images were acquired on a DeltaVision Core system (Applied Precision) using an inverted Olympus IX-71 stand, with an Olympus UPlanSApo ×100 oil immersion objective (numerical aperture (NA) 1.4) and a LED light source. Camera (Photometrics Cool Snap HQ), shutter and stage were controlled through SoftWorx (Applied Precision). Z-series were collected with a spacing of 0.2 μm, and image stacks were subsequently deconvolved in SoftWorx. Immunofluorescence signals in deconvolved images were analysed using ImageJ software (National Institutes of Health, Bethesda, MD). For HEC1 signal quantification, a custom-made macro in ImageJ modified from (Bodor et al. 2014) was used. Intercentromeric distances were measured with ImageJ using multiple z-stack images. The distances between individual kinetochore pairs that were clearly identified in individual z-stacks were measured.

### Immunoblotting analysis

Whole-cell extracts were prepared from HeLa cells transfected with control siRNA, SMC2 siRNA and the indicated vector DNAs. Immunoblotting analysis was performed using the following primary antibodies: rabbit anti-SMC (WCE 1:500) and mouse anti-Tubulin (Sigma B512, 1:10,000). For protein detection and quantification, we used donkey anti-mouse and anti-rabbit fluorescence secondary antibodies (LI-COR Bioscience 1:10,000).

## Results

### PREditOR (protein reading and editing of residues) effectively removes heterochromatin from pericentromeric regions

**Fig. 1:**
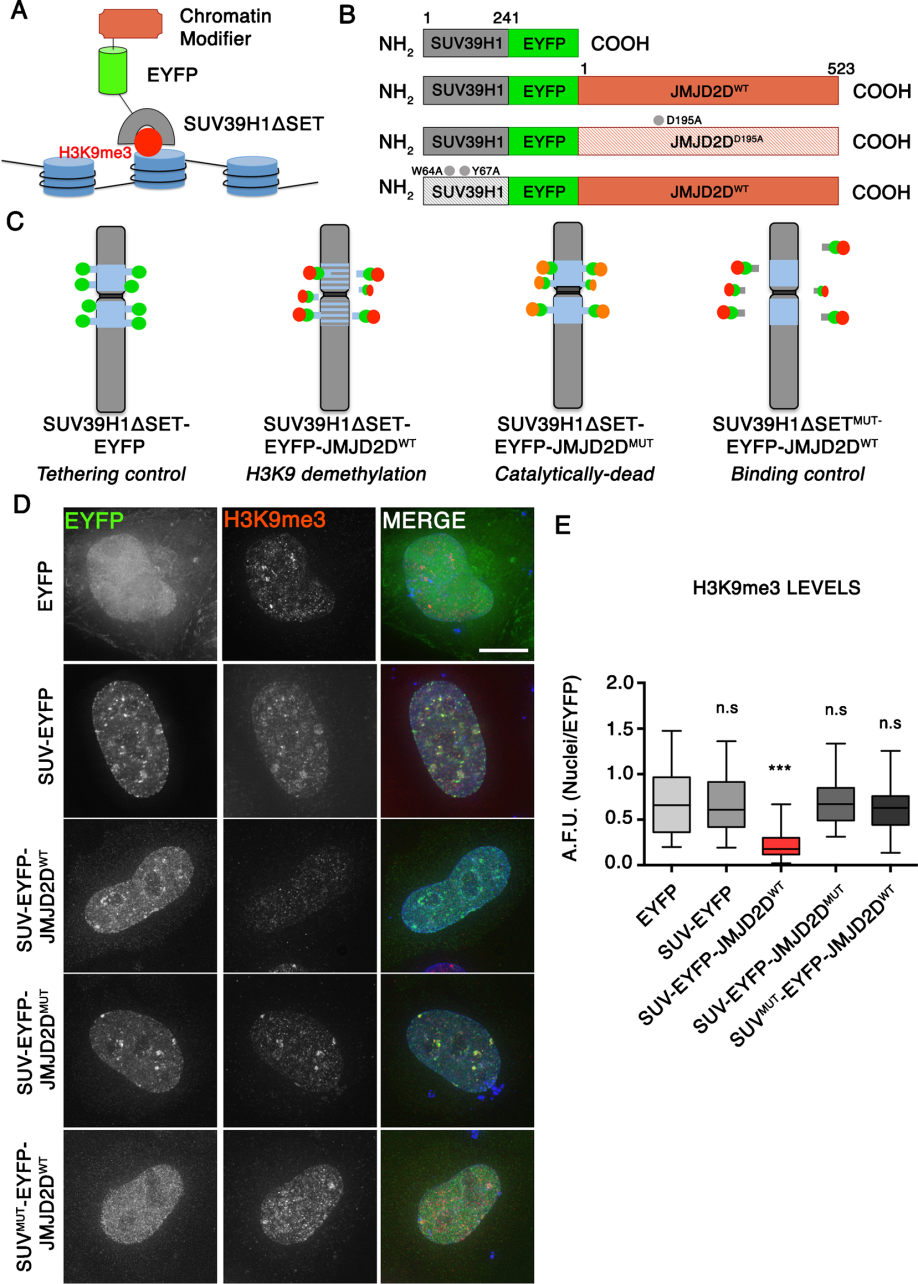
Tethering JMJD2D to heterochromatin decrease H3K9me3 levels. **a** Schematic of the PREdiTOR approach to tether chromatin modifiers to heterochromatin regions. **b** Schematic drawings of the SUV39H1ΔSET-EYFP fusion constructs. **c** Diagram of the experimental design. **d** Representative immunofluorescence images of HeLa cells expressing the indicated SUV39H1ΔSET-EYFP fusion proteins and stained for H3K9me3. *Scale bar* 10 μm. **e** Quantification of fluorescence signals of H3K9me3 staining in individual transfected cells as in d plotted as arbitrary fluorescence units (A.F.U). *Solid bars* indicate the medians of three independent experiments and *error bars* represent the standard error of the mean (s.e.m). *Asterisks* indicate statistical significant differences compared to EYFP (*P < 0.05; **P < 0.001; Student’s *t* test)

To manipulate the epigenetic status of defined chromatin classes, we designed a novel synthetic biology approach that allows us to tether chromatin *E* *ditors* to specific regions of the genome, protein reading and editing of residues (PREditOR). PREditOR is based on the use of fusion proteins consisting of three domains (Supplementary Figure 1a): (i) a *R* *eader* domain that recognizes specific epigenetic modifications, (ii) a fluorescent marker to follow the localization of the fusion protein and (iii) a chromatin *E* *ditor* that functions specifically at or near the tethering site. In order to analyse the role of pericetromeric heterochromatin on chromosome segregation, we fused the N-terminal chromodomain of H3K9-specific methyltransferase SUV39H1 (SUV39H1ΔSET) (a *R* *eader* of H3K9me3) to an EYFP marker (Fig. 1a, b). Removal of the SET domain ensures that this molecule functions solely as a *R* *eader* and not as an enzymatically active *E* *ditor*.

Immunofluorescence analysis after expression of the SUV39H1ΔSET-EYFP fusion protein in HeLa cells showed colocalization with H3K9me3 and CENP-B foci (Fig. 1d and Supplementary Figure 1b). Thus, this fusion protein targets specifically to pericentromeric heterochromatin. SUV39H1ΔSET-EYFP is released from chromatin in early mitosis and rebinds later in anaphase (Supplementary Figure 1c). This is most likely due to a methyl/phos switch effect caused by phosphorylation of histone H3 on Serine 10 catalysed by Aurora B kinase (Fischle et al. 2005; Hirota et al. 2005).

As an *E* *ditor* to remove H3K9me3 from pericentromeric regions, we fused SUV39H1ΔSET-EYFP to the H3K9me3-specific demethylase JMJD2D/KDM4 (SUV39H1ΔSET-EYFP-JMJD2D^WT^) (Fig. 1b, c). Two control molecules were also constructed (Fig. 1b, c). The first was a catalytically dead mutant of JMJD2D carrying a mutation in its jmjC-enzymatic domain fused to SUV39H1ΔSET-EYFP (SUV39H1ΔSET-EYFP-JMJD2D^D195A^). This molecule targets to heterochromatin but cannot demethylate H3K9. The second was a binding-deficient mutant of SUV39H1ΔSET bearing two mutations of its chromatin-binding domain fused to wild type JMJD2D (SUV39H1ΔSET^W61AY67A^-EYFP-JMJD2D^WT^). This molecule has an active demethylase but cannot target specifically to heterochromatin.

Transient expression of SUV39H1ΔSET-EYFP-JMJD2D^WT^ in HeLa cells for 48 h efficiently removed H3K9me3 from pericentromeric loci. Immunofluorescence analysis revealed significantly decreased levels of H3K9me3 levels in cells expressing SUV39H1ΔSET-EYFP-JMJD2D^WT^ compared to the transfection and tethering controls (EYFP and SUV39H1ΔSET-EYFP, respectively) (Fig. 1d, e). Importantly, no differences in H3K9me3 levels were observed after expressing either the catalytically dead mutant (SUV39H1ΔSET-EYFP-JMJD2D^D195A^) or the binding-deficient mutant (SUV39H1ΔSET^W61AY67A^-EYFP-JMJD2D^WT^) (Fig. 1d, e). Apparently, JMJD2D only efficiently demethylates H3K9me3 when it is tethered to heterochromatic regions. Consistent with these results, immunofluorescence staining for HP1α, another hallmark of heterochromatin, revealed a strongly significant decrease in HP1α foci in cells expressing SUV39H1ΔSET-EYFP-JMJD2D^WT^ compared with cells expressing the other control constructs (Supplementary Figure 1d and e).

We also investigated whether chromosomes overall looked more decondensed after expression of SUV39H1ΔSET-EYFP-JMJD2D^WT^ fusion protein. Although there did appear to be some slight decompaction in live images, when chromosomes were fixed and spreads prepared, no significant differences were seen.

We conclude that PREditOR can effectively remove H3K9me3 and specifically disrupt heterochromatin, releasing downstream heterochromatin *R* *eaders* such as HP1α. Importantly, JMJD2D only removes heterochromatin when it is tethered to the pericentromeric regions of chromosomes.

### Heterochromatin removal causes a mitotic accumulation and chromosome segregation defects

**Fig. 2:**
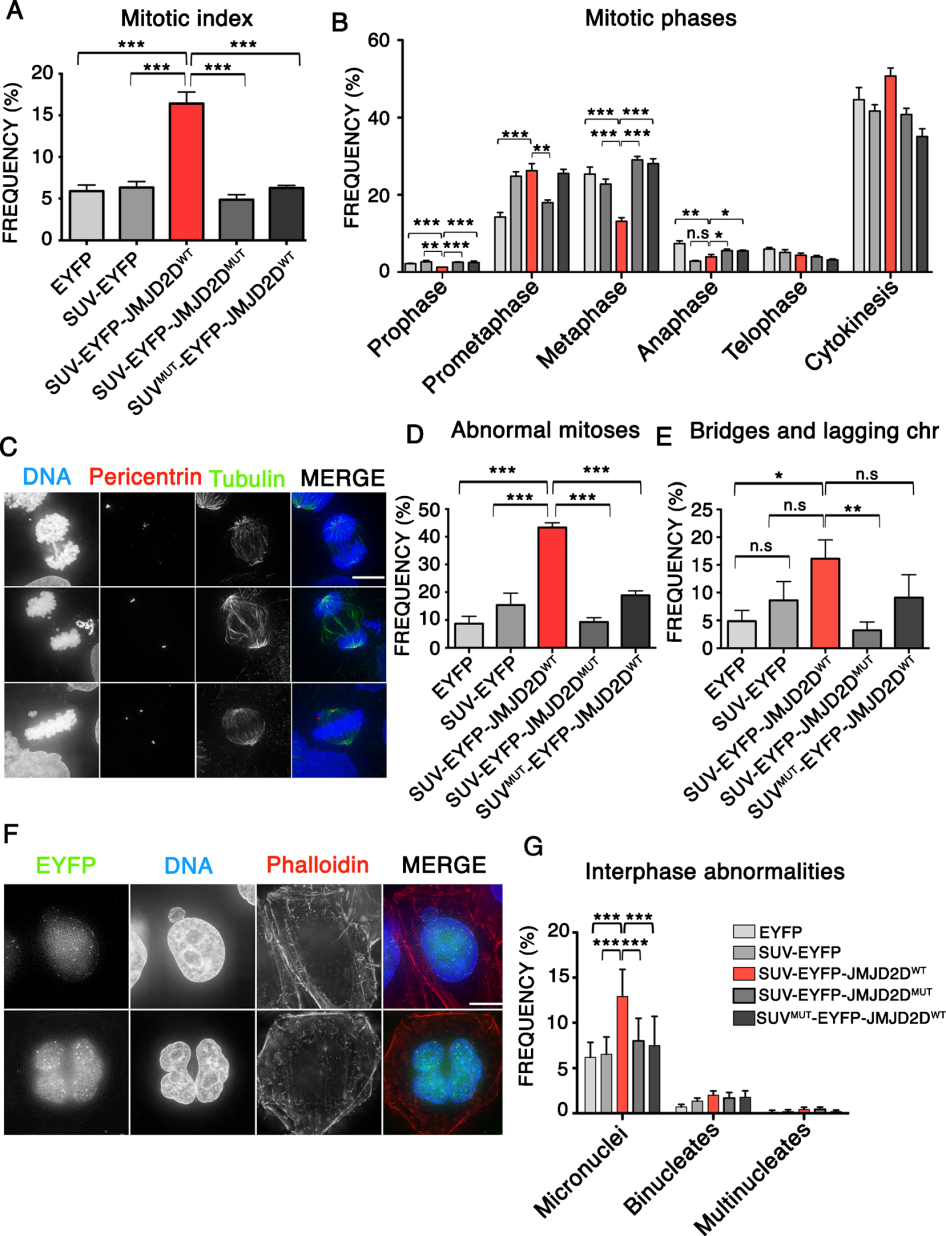
Heterochromatin removal disrupts mitosis and chromosome segregation. **a** Analysis of the frequency of mitotic cells after expressing the indicated SUV39H1ΔSET-EYFP fusion proteins. Data represent the mean and standard error of the mean (s.e.m) of five independent experiments. **b** Analysis of the frequency of every individual mitotic phase in relation of the total number of mitoses. Data represents the mean and the standard error of the mean (s.e.m) of six independent experiments. **c** Representative IF images showing mitotic abnormalities in HeLa cells. Images show examples of chromosome bridges (*top*), lagging chromosomes (*middle*) and uncongressed chromosomes (*bottom*). **d** Analysis of the frequency of abnormal mitoses after expressing the indicated SUV39H1ΔSET-EYFP fusion proteins. Data represent the mean and standard error of the mean (s.e.m) of four independent experiments. **e** Analysis of the frequency of mitotic cells showing bridges or lagging chromosomes after expression of the indicated SUV39H1ΔSET-EYFP fusion proteins. Data represent the mean and standard error of the mean (s.e.m) of four independent experiments. **f** Representative IF images showing interphase abnormalities in HeLa cells. Images show a cell with micronucleus (*top*), and a binucleate cell (*bottom*). **g** Quantification of interphase abnormalities after expressing the indicated SUV39H1ΔSET-EYFP fusion proteins. Data represent the mean and standard error of the mean (s.e.m) of three independent experiments. *Asterisks* indicate statistical significant differences compared to EYFP (*P < 0.05, **P < 0.01, ***P < 0.001; Student’s *t* test)

To analyse the effects of heterochromatin removal on cell division, we expressed the different SUV39H1ΔSET-EYFP fusion proteins in HeLa cells for 48 h and examined their effects on mitosis. Our results show a threefold increase in the mitotic index of cells expressing SUV39H1ΔSET-EYFP-JMJD2D^WT^ compared to cells expressing the control fusion proteins (Fig. 2a). The control results demonstrate that SUV39H1ΔSET-EYFP binding to pericentromeric regions does not interfere with mitotic progression and that the increase in mitotic index is due to the demethylase activity of JMJD2D.

We observed significantly decreased levels of prophase, metaphase and anaphase cells expressing SUV39H1ΔSET-EYFP-JMJD2D^WT^ compared to controls (Fig. 2b). No difference was observed in the frequency of cells in telophase, though a small increase was seen for cells in cytokinesis.

In order to analyse the effects of heterochromatin removal on chromosome segregation, we quantified the frequencies of mitotic abnormalities in HeLa cells expressing the different SUV39H1ΔSET-EYFP fusion proteins. We quantified the frequencies of anaphase bridges, lagging chromosomes, uncongressed chromosomes in metaphase and malformed spindles. Overall, cells expressing SUV39H1ΔSET-EYFP-JMJD2D^WT^ showed a significantly increased frequency of abnormal mitosis compared to cells expressing the other vectors (40 vs 8–15%, respectively) (Fig. 2c, d). In particular, we observed significantly increased frequencies of lagging chromosomes and bridges in cells expressing SUV39H1ΔSET-EYFP-JMJD2D^WT^ (Fig. 2e). Although there was no significant increase in multipolar spindles as judged by pericentrin staining, we did see a high frequency of other spindle malformations (Supplementary Figure 2). Consistent with the increased frequencies of mitotic abnormalities, we also observed significantly increased frequencies of micronuclei, a sensitive reporter for chromosome segregation defects, in interphase cells expressing SUV39H1ΔSET-EYFP-JMJD2D^WT^ compared with controls (13 vs 4–6%) (Fig. 2f, g).

These data suggest that heterochromatin is necessary for correct chromosome segregation during mitosis and that its removal interferes with mitotic progression and chromosome segregation fidelity.

### Perturbing heterochromatin leads to centromere defects

Centromeres direct the assembly the kinetochore, a multi-protein complex that binds to microtubules and directs chromosome segregation (Fukagawa and Earnshaw 2014). However, some kinetochore proteins, including the Mis12 complex, have been reported to bind to the heterochromatin flanking the core centrochromatin (Obuse et al. 2004). In view of the chromosome segregation defects reported above, we asked whether heterochromatin removal is associated with kinetochore defects.

**Fig. 3:**
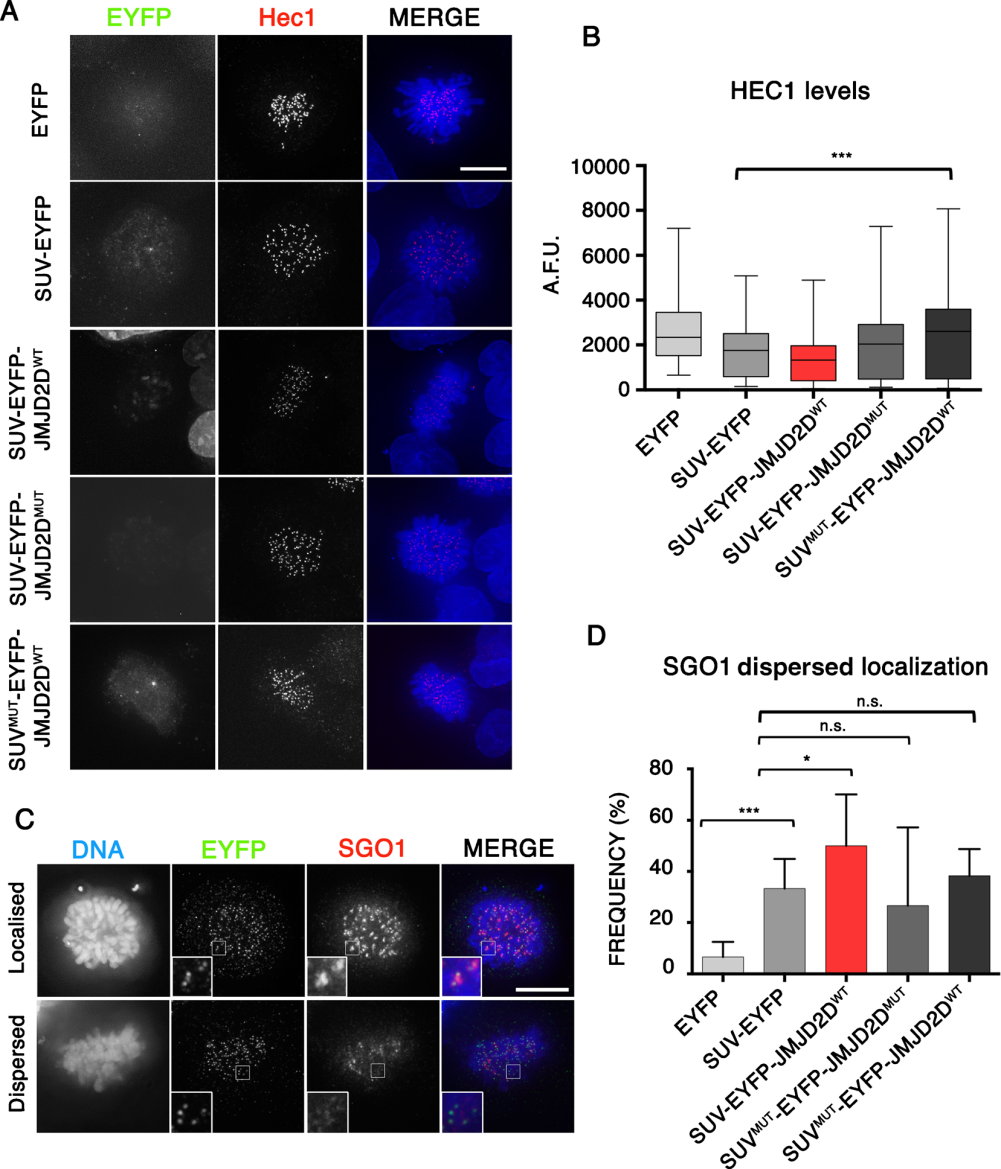
Heterochromatin removal leads to centromere defects. **a** Representative immunofluorescence images of HeLa cells expressing the indicated SUV39H1ΔSET-EYFP fusion proteins and stained for HEC1. *Scale bar* 10 μm. **b** Quantification of fluorescence signals of HEC1 staining in individual cells transfected as in (a) plotted as arbitrary fluorescence units (A.F.U). *Solid bars* indicate the medians of two independent experiments and *error bars* represent the standard error of the mean (s.e.m). **c** Representative immunofluorescence images showing prometaphase cells with localized (*top*) or dispersed (*bottom*) SGO1, using CENP-A as centromere marker. *Scale bar* 10 μm. **d** Analysis of the frequency of cells showing localized or dispersed SGO1 staining after expressing the indicated SUV39H1ΔSET-EYFP fusion proteins. Data represent the mean and standard error of the mean (s.e.m) of three independent experiments

Immunofluorescence staining for the outer kinetochore protein HEC1 was performed after expression of the different SUV39H1ΔSET-EYFP fusion proteins for 48 h (the time point at which we observed significant defects on chromosome segregation). We observed mild but significant decreases in levels of HEC1 in cells expressing all of the SUV39H1ΔSET-EYFP vectors compared to the transfection control (Fig. 3a, b). This suggests that the binding of SUV39H1ΔSET-EYFP alone has an effect on kinetochore structure.

Although all constructs showed statistically significant decreased levels of HEC1 compared with cells expressing EYFP, the greatest decrease was observed in cells expressing SUV39H1ΔSET-EYFP-JMJD2D^WT^ (−49%). Lesser decreases were observed in cells expressing SUV39H1ΔSET-EYFP (−36%), SUV39H1ΔSET-EYFP-JMJD2D^D195A^ (−24%) and SUV39H1ΔSET^W61AY67A^-EYFP-JMJD2D^WT^ (−9%). Therefore, perturbing heterochromatin has a deleterious effect on kinetochore structure. The SUV39H1ΔSET module may exert a dominant-negative effect by competing with *R* *eaders* that bind to H3K9me3. This is consistent with the observation that the SUV39H1ΔSET binding mutant exhibited the mildest phenotype.

Pericentromeric heterochromatin has been associated with the maintenance of cohesin in metaphase (Nonaka et al. 2002). After prophase, cohesin complexes are removed from the chromosome arms, but are retained at centromeres as a result of the activity of Shugoshin 1 (SGO1) (Losada et al. 2002). Given previous links between heterochromatin and cohesin in *S. pombe* (Nonaka et al. 2002), we analysed the localization of SGO1 after expressing the different SUV39H1ΔSET-EYFP fusion proteins in HeLa cells. In transfection controls, SGO1 showed a clear centromeric localization in 95% of the cells (Fig. 3c, d). Expression of the different SUV39H1ΔSET-EYFP proteins resulted in significant increases in the frequency of cells with SGO1 dispersed on chromosome arms (Fig. 3c, d). Thus, SUV39H1ΔSET-EYFP binding to pericentromeric heterochromatin perturbs SGO1 centromeric localization. As was the case for HEC1 staining, cells expressing SUV39H1ΔSET-EYFP-JMJD2D^WT^ more frequently exhibited SGO1 localization defects than did cells expressing other SUV39H1ΔSET-EYFP controls (Fig. 3c, d).

We conclude that SUV39H1ΔSET-EYFP fusion proteins binding to pericentromeres generate mild defects on the kinetochore and SGO1. However, these defects are consistently higher after removing heterochromatin.

### Heterochromatin cooperates with condensin to maintain centromeric stiffness

**Fig. 4:**
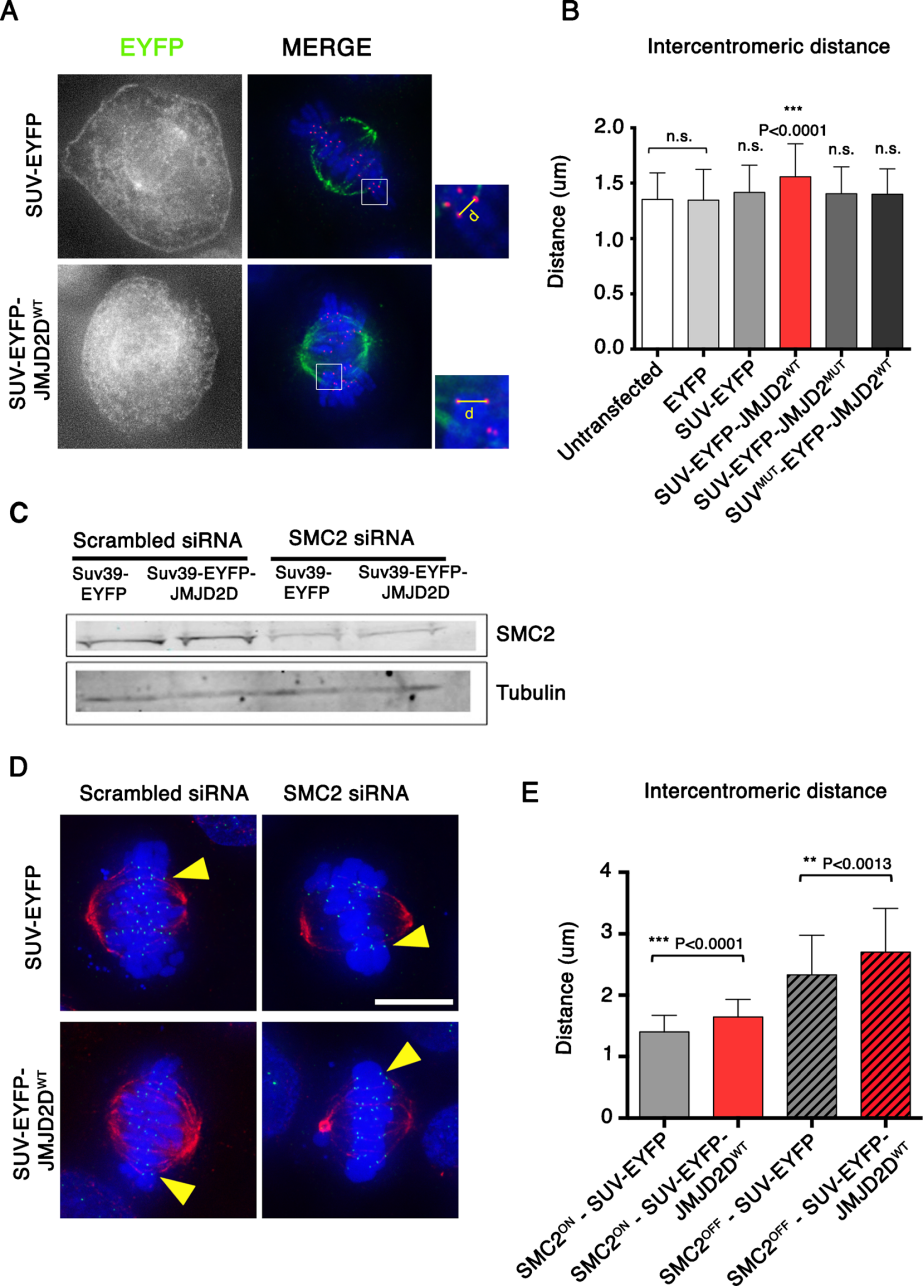
Heterochromatin is necessary to maintain the stiffness of the centromere in metaphase. **a** Representative immunofluorescence images of HeLa cells expressing the indicated SUV39H1ΔSET-EYFP fusion proteins and stained for CENP-C and Tubulin. **b** Quantification of intercentromeric distances in chromosomes under tension after expressing the indicated SUV39H1ΔSET-EYFP fusion proteins. Data represent the mean and standard error of the mean (s.e.m) of three independent experiments. **c** Immunoblot of whole HeLa cell protein extract transfected with the indicated siRNA and DNAs. Immunoblot for SMC2 with Tubulin as a loading control. **d** Representative immunofluorescence images of HeLa cells expressing the indicated SUV39H1ΔSET-EYFP fusion proteins and transfected with the indicated siRNA. **e** Quantification of intercentromeric distances in chromosomes under tension after expressing the indicated SUV39H1ΔSET-EYFP fusion proteins and siRNAs. Data represent the mean and standard error of the mean (s.e.m) of three independent experiments. *Asterisks* indicate statistical significant differences compared to EYFP (*P < 0.05, **P < 0.01, ***P < 0.001; Student’s *t* test)

We and others previously showed that the condensin complex is important for maintaining the rigidity of the centromere (Gerlich et al. 2006; Ribeiro et al. 2009; Jaqaman et al. 2010). We hypothesized that condensin might act by regulating the compliance of centromeric heterochromatin (Ribeiro et al. 2009). To test the effect of removing heterochromatin on centromere stiffness, we expressed the various SUV39H1ΔSET-EYFP fusion proteins in HeLa cells for 48 h and analysed the distances between sister kinetochores on metaphase chromosomes. We observed a significant increase in this distance after expressing SUV39H1ΔSET-EYFP-JMJD2D^WT^ compared with controls (Fig. 4a, b). This supports the notion that pericentromeric heterochromatin has a role in maintaining centromeric stiffness.

In order to investigate our hypothesis that there is an interaction between condensin and heterochromatin in maintaining centromeric stiffness, we partly depleted SMC2 in HeLa cells using published siRNAs (Gerlich et al. 2006). Western blot analysis showed a 61% decrease in SMC2 levels after siRNA transfection (Supplementary Figure 3a). This was confirmed by immunofluorescence analysis, which showed a reduction of SMC2 levels on chromosomes compared with the control siRNA (Supplementary Figure 3b). Although 39% of the SMC2 remained in cells under these conditions, we observed the characteristic phenotypes of condensin-depleted cells, including dramatic changes in chromosome morphology, increased frequencies of lagging chromosomes and chromosome bridges (Supplementary Figure 3b and c).

Once the conditions for SMC2 depletion with siRNA were established, we analysed the intercentromeric distances of metaphase chromosomes after expressing either SUV39H1ΔSET-EYFP or SUV39H1ΔSET-EYFP-JMJD2D^WT^ in the presence or absence of SMC2 depletion (Fig. 4c). Consistent with previous results from our group (Ribeiro et al. 2009), we observed a strong increase in intercentromeric distances in cells depleted of SMC2 compared with those transfected with the control siRNA (Fig. 4d, e). Strikingly, our analysis showed further significant increases of intercentromeric distances in cells expressing SUV39H1ΔSET-EYFP-JMJD2D^WT^ compared with controls expressing SUV39H1ΔSET-EYFP. This additional effect upon removal of heterochromatin was seen both in the presence and absence of SMC2 (Fig. 4d, e).

These results show that heterochromatin cooperates with condensin to maintain centromeric stiffness. However, the additive nature of the observed effect suggests that condensin and heterochromatin make at least partly independent contributions.

### Heterochromatin is essential for proper chromosome passenger complex localization

**Fig. 5:**
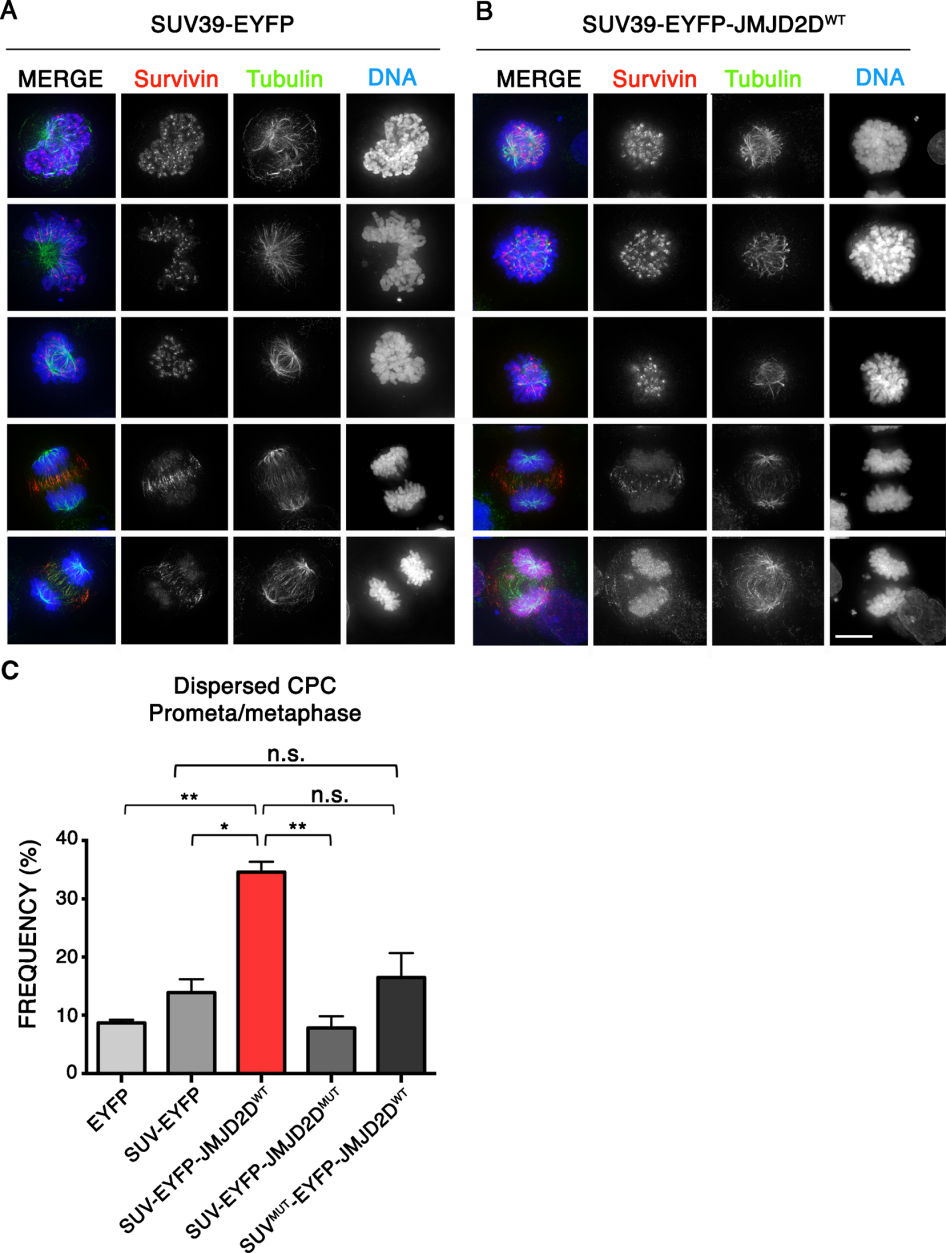
Heterochromatin removal disrupts chromosomal passenger localization in mitosis. **a**, **b** Representative immunofluorescence images of HeLa cells expressing SUV39H1ΔSET-EYFP (**a**) or SUV39H1ΔSET-EYFP-JMJD2D^WT^ (**b**) fusion protein and stained for Survivin and Tubulin. *Scale bar* 10 μm. **c** Analysis of the frequency of cells showing dispersed CPC in prometaphase and metaphase after expressing the indicated SUV39H1ΔSET fusion proteins. Data represent the mean and standard error of the mean (s.e.m) of two independent experiments. *Asterisks* indicate statistical significant differences compared to EYFP (*P < 0.05, **P < 0.01; Student’s *t* test)

The chromosome passenger complex (CPC) of Survivin, INCENP, Borealin and its catalytic subunit Aurora B Kinase localizes to different targets during mitosis, where it regulates key mitotic events (Carmena et al. 2012). In early mitosis, the CPC is localized at inner centromeres, where it ensures that kinetochore-microtubule attachments are correct and regulates the spindle assembly checkpoint. During anaphase, it transfers to the midzone where it regulates the completion of cytokinesis (Fig. 5a) (Carmena et al. 2012).

It has been reported that centromeric HP1 targets the CPC to centromeres in early mitosis (Ainsztein et al. 1998; Liu et al. 2014). In order to study the role of heterochromatin on CPC localization at centromeres, we expressed the different SUV39H1ΔSET-EYFP vectors in HeLa cells for 48 h and analysed the localization of the CPC by staining for Survivin (Fig. 5a, b). In control cells expressing SUV39H1ΔSET-EYFP, the CPC concentrates at centromeres during prometaphase (Fig. 5a, c). Strikingly, our immunofluorescence analysis of cells expressing SUV39H1ΔSET-EYFP-JMJD2D^WT^ showed an increased frequency of cells with the CPC dispersed on the chromosome arms in early mitosis (Fig. 5b, c). Moreover, we observed defects in CPC transfer to the midzone in late mitosis (Fig. 5a, b, bottom panels). Expression of SUV39H1ΔSET-EYFP-JMJD2D^WT^ led to an increased frequency of cells in late mitosis in which the CPC remained attached to chromosomes and failed to concentrate at the spindle midzone.

We conclude that heterochromatin is necessary for efficient CPC localization at centromeres and also for its transfer to the midzone in late mitosis.

## Discussion

The ever-expanding panoply of histone modifications function by influencing the overall structure of chromatin and by regulating the binding of chromatin *R* *eaders* (Bannister and Kouzarides 2011). Epigenetics can be thought of as functioning through Editor → Mark → Reader → Chromatin state (E → M → R → C) pathways that can be artificially engineered by our synthetic biology approach PREditOR. Three examples amongst the many *R* *eaders* that bind to specific histone modifications include the chromatin remodeller CHD1, which binds to H3K4me2/3 (Sims et al. 2005), the polycomb repressor complex subunit PRC1, which binds to H3K27me3 (Cao et al. 2002; Levine et al. 2002) and the H3K9 methyltransferase SUV39H1, which binds to H3K9me2/3 (Rea et al. 2000). Our PREditOR approach can be applied to study any of these pathways as we have shown in several previous studies focused on the epigenetic landscape of the centromere of a synthetic human artificial chromosome (HAC – (Nakano et al. 2008; Cardinale et al. 2009; Bergmann et al. 2011; Martins et al. 2016; Molina et al., 2016).

Here, we describe the first use of PREditOR to study native human chromosomes. We designed a specific PREditOR strategy to tether an Editor to constitutive heterochromatin regions as a fusion to the chromodomain of the H3K9-specific methyltransferase SUV39H1 linked to EYFP. It was previously reported that the truncated SUV39H1 protein maintaining only its chromodomain targets specifically to pericentromeric regions (Melcher et al. 2000; Krouwels et al. 2005), which comprise the bulk of constitutive heterochromatin in eukaryotic cells. Consistent with those reports, we observed a strong colocalization of SUV39H1ΔSET-EYFP with heterochromatin foci containing the centromeric protein CENP-B in HeLa cells. We also observed a similar colocalization after expressing SUV39H1ΔSET-EYFP in other human cell lines, including HT1080 and RPE1hTERT (data not shown). Immunofluorescence analysis staining for H3K9me3 and HP1α after expressing SUV39H1ΔSET-EYFP-JMJD2D^WT^ showed residual diffuse antibody signals in all nuclei, but a loss of the characteristic heterochromatin foci (Fig. 1d and Supplementary Figure 1d). These results suggest that SUV39H1ΔSET-EYFP expression disrupts pericentromeric heterochromatin.

Removal of heterochromatin leads to defects in mitosis subsequent to delays in prometaphase. Many of these may be explained by defects in localization of the CPC observed after heterochromatin removal (Fig. 5). The CPC controls the proper attachment of kinetochores to microtubules and activates the spindle assembly checkpoint until chromosomes are properly aligned (Carmena et al., 2012).

Importantly, cells expressing several control SUV39H1ΔSET-EYFP fusion proteins behaved like the transfection control (EYFP alone). Thus, SUV39H1ΔSET binding did not interfere with normal mitotic progression. In contrast, other authors have observed a mitotic arrest after inhibiting SUV39H1 by its specific inhibitor chaetonin, which decreases the levels of heterochromatin (Chu et al. 2014; Chu et al. 2012). This stronger phenotype could be due to off-target effects of the drug or to the action of SUV39H1 on other targets required for mitotic progression.

Disruption of pericentromeric heterochromatin has been reported to affect chromosome segregation and to increase genomic instability in yeast (Ekwall et al. 1995), mice (Peters et al. 2001) and human cells (Slee et al. 2012). Surprisingly, although double null mutants for Suv39h1 and Suv39h2 in mice show severely impaired viability, a low level of live offspring (33%) was obtained. These double null or Suv39h1-null mice showed an increased predisposition to B cell lymphomas with hyperdiploid karyotypes, suggesting that lack of pericentromeric heterochromatin might increase genomic instability by impairing chromosome segregation (Peters et al. 2001). Consistent with these results, our data show a threefold increase on chromosome segregation defects after removing heterochromatin by expressing SUV39H1ΔSET-EYFP-JMJD2D^WT^ in human cells for 48 h. Since HeLa cells divide approximately once every 24 h, chromosome segregation defects must occur in the first or at most the second mitosis after heterochromatin removal.

Heterochromatin removal appeared to affect several protein complexes that are important for centromere function. Two of these effects were expected. First, we observed that heterochromatin depletion causes an increase in the compliance (stretchiness) of inner centromeres, with the result that the distance between sister kinetochores is increased. This is consistent with previous suggestions that condensin regulates centromeric stiffness by regulating the compliance of centromeric heterochromatin (Ribeiro et al. 2009; Jaqaman et al. 2010). It is not clear if this effect is due to changes in the higher-order packing of the chromatin fibre or is mediated by centromeric cohesin complexes, as heterochromatin removal resulted in SGO1 displacement, which might result in lower levels of cohesin at the centromere. The association of heterochromatin with cohesin is currently a matter of debate (Bernard et al. 2001; Koch et al. 2008). Since SGO1 is involved in cohesin complex maintenance at centromeres after prophase until anaphase onset, our results suggest that interfering with SGO1 targeting to centromeres caused by heterochromatin removal might perturb sister chromatid cohesion.

We also observed that centromeric heterochromatin is required for efficient localization of the chromosomal passenger complex (CPC) to inner centromeres during early mitosis. This could be predicted from results indicating that binding to SGO1 (Yamagishi et al. 2010) and to heterochromatin protein HP1α are both important for targeting of the CPC in early mitosis. As stated above, defects in CPC localization may explain many of the mitotic defects observed following heterochromatin removal.

Binding of SUV39H1ΔSET fusion proteins to pericentromeric heterochromatin had a small, but reproducible effect on assembly of the kinetochore. This was not expected, because the kinetochore assembles on the surface of CENP-A-containing centrochromatin and not on the pericentromere. Indeed, in preliminary results, we noticed an increase in the amount of CENP-A associated with centromeres when heterochromatin was depleted. This is consistent with previous results in which the Rb pathway was perturbed, leading to a decrease in H3K9 methylation (Sullivan et al. 2011). SUV39H1 has a dual role in heterochromatin formation and maintenance: it acts as a methyltransferase and also has a structural role at pericentromeric heterochromatin by binding HP1α (Haldar et al. 2011). Overexpressed SUV39H1ΔSET might affect kinetochore assembly either by perturbing HP1α dynamics, or possibly as a result of its effects on SGO1 or CPC localization.

The present results demonstrate the utility of the PREditOR approach for epigenetic engineering—in this case to specifically remove heterochromatin from dividing cells. It had been widely assumed that heterochromatin would be important for mitotic chromosome segregation, but previous studies were plagued by the possibility of off-target effects of drugs and genetic manipulations. Here, we confirm that heterochromatin is indeed important for mitotic chromosome segregation, although the effects of heterochromatin removal on pericentromeric heterochromatin structure, mitotic progression and on chromosome segregation are remarkably mild. We conclude that heterochromatin at centromeres is necessary to maintain genomic stability.

## Electronic supplementary material

Figure S1. Tethering JMJD2D to heterochromatin disrupt heterochromatin structure. (Related to Fig. 1). **(a)** Schematic of the vectors used for PREditOR approach. **(b)** Representative image of a HeLa cell expressing SUV39H1ΔSET –EYFP and immunostained with CENP-B. **(c)** Representative IF images showing the localization of SUV39H1ΔSET –EYFP fusion protein in different phases of mitosis. Scale bar: 10 μm. **(d)** Representative IF images of HeLa cells expressing the indicated SUV39H1ΔSET-EYFP fusion proteins and staining for HP1α. Scale bar: 10 μm. **(e)** Quantification of the number of f HP1α foci in individual cells transfected as in b plotted. Solid bars indicate the medians of three independent experiments and error bars represent the standard error of the mean (s.e.m). Asterisks indicate statistical significant differences compared to EYFP (*** *P* < 0.001; t-student test). (PSD 21741 kb)

Figure S2. Heterochromatin removal does not change the number of spindle poles. (Related to Fig. 2). **(a)** Representative IF images staining for pericentrin and Tubulin showing normal (top) and abnormal polar numbers as judged by pericentrin foci. **(b)** Quantification of cells showing abnormal number of pericentrin foci after expressing the indicated SUV39H1ΔSET-EYFP fusion proteins. Data represent the mean and standard error of the mean (s.e.m) of three independent experiments. (PSD 15094 kb)

Figure S3. Knockdown of SMC2 by siRNA in HeLa cells. (Related to Fig. 4). **(a)** Immunoblot of whole HeLa cell protein extract transfected with the indicated siRNA at the indicated concentrations. Immunoblot for SMC2 with Tubulin as a loading control. **(b)** Representative IF images staining for SMC2, Tubulin and ACA in cells transfected with a control siRNA (top) and with siRNA for SMC2 (bottom). **(c)** Quantification of the frequency of normal and abnormal mitosis in HeLa cells transfected with the indicated siRNAs at the indicated concentrations. (PSD 8891 kb)
